# The leading role of pathology in assessing the somatic molecular alterations of cancer: Position Paper of the European Society of Pathology

**DOI:** 10.1007/s00428-020-02757-0

**Published:** 2020-03-02

**Authors:** Xavier Matias-Guiu, Giorgio Stanta, Fátima Carneiro, Ales Ryska, Gerald Hoefler, Holger Moch, on behalf of the European Society of Pathology (ESP)

**Affiliations:** 1 Hospital Universitari Arnau de Vilanova. Universitat de Lleida, IRBLleida. CIBERONC, Hospital U de Bellvitge. IDIBELL, University of Barcelona, Av Rovira Roure, 80, 25198 Lleida, Spain; 2 Department of Medical Sciences, University of Trieste, Trieste, Italy; 3 Department of Pathology, Medical Faculty of the University of Porto/Centro Hospitalar Universitário São João and Ipatimup/i3S, Porto, Portugal; 4 The Fingerland Department of Pathology, Charles University Medical Faculty and University Hospital, Hradec Kralove, Czech Republic; 5 Diagnostic and Research Institute of Pathology, D&R Center of Molecular BioMedicine, Medical University of Graz, Graz, Austria; 6 Institute for Pathology and Molecular Pathology, University Hospital Zurich, Zurich, Switzerland

**Keywords:** Molecular pathology, Pathology, Pre-analytical issues, Integrative pathology

## Abstract

Molecular pathology is an essential part of pathology complementing conventional morphological tools to obtain a correct integrated diagnosis with appropriate assessment of prognosis and prediction of response to therapy, particularly in cancer. There is a concern about the situation of molecular pathology in some areas of Europe, namely, regarding the central role of pathologists in assessing somatic genomic alterations in cancer. In some countries, there are attempts that other laboratory medicine specialists perform the molecular analysis of somatic alterations in cancer, particularly now when next generation sequencing (NGS) is incorporated into clinical practice. In this scenario, pathologists may play just the role of “tissue providers,” and other specialists may take the lead in molecular analysis. Geneticists and laboratory medicine specialists have all background and skills to perform genetic analysis of germline alterations in hereditary disorders, including familial forms of cancers. However, interpretation of somatic alterations of cancer belongs to the specific scientific domain of pathology. Pathologists are necessary to guarantee the quality of the results, for several reasons: (1) The identified molecular alterations should be interpreted in the appropriate morphologic context, since most of them are context-specific; (2) pre-analytical issues must be taken into consideration; (3) it is crucial to check the proportion of tumor cells in the sample subjected to analysis and presence of inflammatory infiltrate and necrosis should be monitored; and 4) the role of pathologists is crucial to select the most appropriate methods and to control the turnaround time in which the molecular results are delivered in the context of an integrated diagnosis. Obviously, there is the possibility of having core facilities for NGS in a hospital to perform the sequence analysis that are open to other specialties (microbiologists, geneticists), but also in this scenario, pathologists should have the lead in assessing somatic alterations of cancer. In this article, we emphasize the importance of interpreting somatic molecular alterations of the tumors in the context of morphology. In this Position Paper of the European Society of Pathology, we strongly support a central role of pathology departments in the process of analysis and interpretation of somatic molecular alterations in cancer.

## Cancer as a complex genetic and epigenetic disease

Cancer is a complex disease. Hereditary and environmental factors are important in its development. Cancer results from accumulation of genetic and epigenetic alterations in cells [1], under the influence of the immune system and the non-neoplastic microenvironment, which is usually tumor-type specific. Cancer cells have important interactions with non-cancer host cell populations, such as fibroblasts, vessels, or inflammatory cells [2].

Every type of cancer has its own molecular profile. Even more, each individual cancer in a particular patient has its unique repertoire of molecular abnormalities [3]. With the exception of some types of tumors, it is virtually impossible to find two tumors from two different patients, even from the same organ, having the same genetic and epigenetic background. Thus, from the biological viewpoint, each tumor represents a unique scenario resulting from a sequence of individual molecular changes. We know today that the morphologic appearance of the tumors reflects the genetic and epigenetic changes present in tumor cells. Microscopy has been crucial in recognizing biologically distinct entities with specific molecular features, and, on the other hand, molecular profiling has allowed identifying new tumor types that turned out to have specific microscopic features.

## Role of pathology in the diagnosis of cancer

For many years, cancer has been diagnosed on the basis of its microscopic appearance. Several decades ago, identification of proteins by immunohistochemistry became an important tool in cancer diagnosis [4]. Later on, identification of single gene alterations (mutations, gene fusions, amplifications, by PCR-based mutation analysis, or FISH) was incorporated into pathological report and is used today as a tool to help in diagnosis, prognosis, and prediction in response to treatment. Over the years, WHO classifications of tumors have transformed from exclusive morphologic criteria into integrated morphologic-molecular schemes. Novel technologies like NGS are providing more information to understand the molecular profile of each particular tumor. Bioinformatic support is an essential tool for the analysis of large-scale molecular datasets. Microscopic appearance, nevertheless, provides the framework in which molecular analysis should be interpreted.

Pathology is the medical discipline responsible for the diagnosis of diseases based on the morphological appearance under the microscope of tissue or cytological samples. Cancer is one of the areas where the progress in pathology is most dynamic and significant. The role of pathologists in diagnosis of cancer, as well as assessment of prognostic and predictive factors, is crucial. The task for pathologists is not only the evaluation of the gross appearance of the lesion together with microscopic assessment of neoplastic tissue. To guarantee high reliability of pathological diagnosis, the so called pre-analytical conditions (handling of the material before the testing itself) must be controlled. Another crucial issue is the appropriate sampling of most relevant diagnostic areas, as well as selection of the most informative tumor areas for additional ancillary techniques [5, 6].

Generally speaking, a patient does not have a proven cancer diagnosis, until a pathologist establishes such diagnosis in a histologic or cytologic specimen. Obviously, there are few exceptions to this statement, for some malignancies and disseminated tumors, in which obtaining a tissue sample may be too risky for the patient.

Pathologists are not laboratory specialists who identify, quantify, and report molecules from blood or other biological samples. We are medical specialists interpreting the microscopic appearance of the tumor in the setting of the molecular, clinical, and imaging features. Occasionally, similar microscopic pictures can lead to different diagnoses, depending on age, tumor site, or clinical scenario.

Interpretation by pathologists is also essential for correct interpretation of molecular research data resulting from the analysis of tissues and cells [7, 8]. The anomaly of interpreting and reporting research pathologic features in human and animal models without the appropriate expertise in pathology has led to inadequate interpretation and occasional retraction of erroneous results, even in highly prestigious peer-review journals [9]. The term “pathology by yourself” or “do it yourself” has been used to designate this incorrect way of interpreting molecular data, in which researchers perform their own pathologic analysis, lacking appropriate training in pathology [10]. It would be inadmissible to accept similar practice when providing pathologic diagnosis in patients’ tissue samples (biopsies and/or surgical specimens), potentially resulting in inappropriate or even entirely wrong decisions in patients’ care.

## Germline molecular alterations in cancer

The genetic alterations that are detected in cancer tissues can be germline or somatic. Cancer-related germline genetic abnormalities occur in the setting of clinical situations with an increased risk for the development of cancer. Familial cancer syndromes are the paradigmatic examples. Germline alterations are important for tumor development, and their identification may be important for prognosis or even for prediction of response to certain types of systemic anticancer treatment. Germline alterations are detected in tumor tissue but also in normal non-neoplastic tissues from cancer patients. Peripheral blood is the most frequently used biological material for identification of germline changes. Management of DNA and RNA from peripheral blood is less challenging than that obtained from tumor tissue, as the nucleic acids in the blood are less negatively influenced by pre-analytical conditions.

Identification and interpretation of germline alterations in cancer do not usually require integration of the results in the context of morphological features of the tumors. It may be perfectly performed by geneticists, who have the appropriate expertise, including skills to classify the identified variations regarding their impact and distinguish between pathogenic changes from those that are not. Bioinformatic support is essential. Since the tumors occurring in the setting of familial cancer syndromes may sometimes have specific morphologic features, it is desirable to have pathologists as part of the multidisciplinary teams interpreting results of germline molecular testing and establishing screening or surveillance strategies. However, it does not seem necessary that pathologists have the lead in these clinical strategies.

Occasionally, analysis of somatic mutations may lead to identification of a genetic change that may be suspicious to be in fact a germline alteration. Additional conventional germline mutation analysis (from peripheral blood) in close collaboration with clinical geneticist may be mandatory to confirm the germline nature of these genetic variants.

Evidence of *BRCA1/2* mutations or microsatellite instability (Lynch syndrome) should be noted in the pathology report with a recommendation that clinical geneticists have to be involved to perform genetic counseling of the patient with this family.

## Importance of interpreting somatic alterations in cancer in the appropriate morphological context

The scenario of somatic genomic alterations of cancer is different from that of the germline ones. The clinical and pathologic contexts are important. Integration of molecular results with microscopic features is necessary. There are tissue-specific differences in tumorigenesis and the organization of individual oncogenic signaling pathways. There are many examples of somatic alterations (*BRAF*, *KIT*) that have different significance depending on tumor type [11]. There are many evidences showing that the genomic landscape and the relevance of activated signaling pathways differ with respect to tumor type and organ location [12]. Different cells and tissues have important differences in their response to oncogenic driver mutations [13, 14], and cancer drivers may have different roles in different cell types or stages of differentiation. Recent basket trials provide evidence that the response to a molecular alteration-specific anticancer drug often depends on the pathologic cancer type as well as on the tissue of origin [11]. The context-specific preservation of feedback explains why and how oncogene addiction maintains a certain level of signaling output and counteracting intrinsic feedback inhibition [15], depending on cell type.

Interpretation of somatic alterations of cancer should be therefore performed in the setting of pathology departments. In this regard, it is necessary that pathology departments incorporate specialists with strong molecular expertise (biochemists, molecular biologists) as well as bioinformaticians, who should be integrated as full-fledged staff members. This is currently the situation in a significant proportion of tertiary hospitals across Europe; however, in some areas, there are administrative difficulties for an efficient incorporation.

Incorporation of NGS into clinical practice is having a tremendous impact in management of cancer patients [16]. In some scenarios, single-gene approaches seem to be still cost-effective, but in steadily growing proportion of clinical situations, there is a need for multigene approach, i.e., analyzing sets of multiple clinically relevant genes at the same time. This strategy is saving the tissue, which is often limited, increasing cost-efficacy, and reducing turnaround time of response. It is the responsibility of pathologist to select, on the basis of morphologic findings, the markers which should be tested and to guarantee the choice of appropriate methods as well as optimal use of the limited tissue sample. While NGS is frequently used for identification of germline alterations in cancer, because a high number of simultaneously analyzed genes are usually necessary, its use to identify somatic abnormalities is also becoming more accessible.

In some centers, NGS equipment is based in pathology departments. In other centers, NGS facility is shared in central core facilities, available to several different specialties. In some of these centers, pathologists have access to NGS equipment and perform somatic molecular interpretation followed by delivery of an integrated pathologic report. In other centers, NGS is performed by other specialists, and pathologists are only requested to simply provide tissue samples, without too much interaction, and insufficient involvement in reporting the results. We are strongly convinced that this role of just “tissue providers” would be totally inappropriate, carrying important risks of incorrect interpretation of molecular findings outside of the tissue context, and might be thus potentially detrimental to patient safety.

Sometimes, hospital administrators do not consider the abovementioned conditions and take unfortunate decisions allowing molecular testing and interpretation of cancer somatic alterations in facilities, outside pathology departments, without guaranteeing integration into the appropriate pathologic context. These wrong decisions are usually based on economical or organizational parameters, not supported by scientific evidence.

Another important fact which must be taken into consideration is the issue of tumor heterogeneity. There is increasing evidence that tumors are composed of subpopulations of cells with distinct genetic alterations [17, 18]. Tumor heterogeneity has its consequences often also at the microscopic level. With the advent of NGS techniques, the occurrence and extent of intra-tumor heterogeneity is becoming acknowledged and correlates many times with different microscopic features. Spatial and temporal heterogeneity may permit the tumor to adapt to variations in tumor microenvironment. Comparison of the somatic mutations of primary tumors and metastasis or in tumor areas with different histological patterns indicates that somatic gene alterations may vary substantially. It has been shown that tumor heterogeneity can be a challenge for evaluating the suitability of individual targeted therapies, since abnormalities in target genes can be heterogeneously distributed in different subpopulations of an individual tumor. Evaluation of tumor heterogeneity at the microscopic level in conjunction with its tumor-associated microenvironment is necessary for the optimal selection of a tissue block for molecular analysis; this step, seemingly simple, may have a crucial impact on assessing correctly prognosis or prediction of response to treatment.

As mentioned before, the clinical and microscopic context is essential to interpret appropriately the results. This is relevant not only for tumor morphology but also to interpret the role of non-cancer microenvironment in conjunction with molecular data. However, it is important to emphasize that any molecular test is just one small part of the entire complex pathologic diagnosis. Traditional pathological features, such as gross findings, tumor stage, histological type, and grade based on microscopic evaluation and molecular findings, should be therefore integrated in a combined pathologic report. Moreover, digital pathology, which is recently being implemented in many pathology departments, may become an additional useful tool to integrate molecular results in the appropriate pathological context [19]. Advances in digital pathology will help in implementing algorithms of artificial intelligence, as an additional tool for integrating all information retrieved from a cancer tissue specimen [20].

**Fig. 1:**
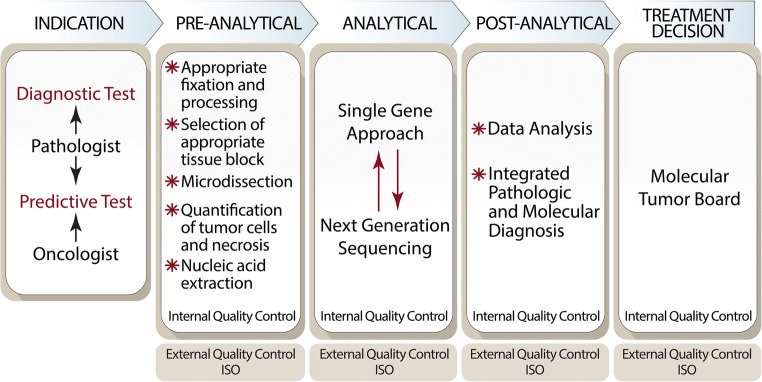
Workflow diagram of somatic molecular testing

Indications for molecular analysis in pathology have changed over the last decade. Molecular pathology was initially performed virtually exclusively for diagnostic purposes in hematologic disorders and solid tumors, and pathologists were the solely responsible for deciding when and which specific test should be performed in a particular case. Nowadays, however, a significant proportion of the molecular tests are performed for predictive purposes, to identify subsets of cancer patients who will have higher probability to respond to certain anticancer treatment. This has led to a change of roles, and the tests are now frequently requested by clinician. Under such circumstances, a close interaction between oncologists and pathologists is necessary, with efficient workflow and decision-making strategies, in which Molecular Tumor Boards are excellent scenarios. Nowadays, many pathology departments organize Molecular Tumor Boards, involving molecular biologists, bioinformaticians, and oncologists, to discuss the relevance of NGS findings for targeted therapies in different organ tumors (lung, breast, urological cancers) together. These Molecular Tumor Boards represent ideal platforms for a comprehensive discussion of all aspects of molecular diagnostics and the consequences for targeted therapies. It should be emphasized, however, that pathologist should play a role in the decision about the most appropriate molecular assay that must be performed, particularly for cancer diagnosis (Fig. 1).

The number of molecular tests that are incorporated to regular testing of tissues is constantly growing, in concordance with development of novel targeted therapies requiring new predictive biomarkers [21]. In this scenario, pharmaceutical companies involved in the development of innovative treatment are becoming new actors, and their important role has to be taken into account. However, this is a two-side process. Recent experience from development of new checkpoint inhibitors clearly demonstrates that pathologists should be involved also in the planning of new clinical trials, regarding eligibility, stratification, quality control, and particularly parameters for evaluation of response to therapy, and establishment of criteria for scoring predictive biomarkers. Only so we can eliminate (or at least minimize) later complications and issues during the implementation of testing after the drug approval.

Currently, a new paradigm for cancer care is emerging, where treatments are identified based on the specific genetic profile of an individual’s tumor, regardless of where it first originated. Pathologists diagnose tumors based on a specific histological type and the organ of origin. However, organizations such as the American Food and Drug Administration (FDA) have recently shown a willingness to accept novel trial designs—such as basket studies, which enroll patients for treatments based on the biology of their tumor, not their tumor type. These “tumor-agnostic” agents target gene alterations thought to drive tumor growth regardless of cancer histology. “Tumor-agnostic” therapies are based on identification of microsatellite instability or high tumor mutational burden for immuno-oncological therapies [22]. Other examples include TRK inhibitors, showing clinically meaningful responses in patients with NTRK fusions. NTRK gene fusions are present in a broad range of more than 20 different tumor types, including those of the head and neck, salivary gland, bladder, and lung [23, 24]. It is the task of pathologists to proof this controversial concept and to evaluate such tumor-agnostic treatments. It is currently not clear to what extent tumor-agnostic therapies will be implemented at the long term and, also, whether they will be limited to a few drugs or extended to a high number of agents. Part of the pathology community is skeptical, based on the specificities of signaling pathway activation regarding histological type and organ of origin. It is evident, however, that pathologists should be ready to incorporate biomarker identification of these agnostic therapy-related markers that will probably coexist in the future with tumor-specific predictive biomarkers. Even in this scenario, interpretation of molecular findings should be done in the setting of microscopical appearance and control of pre-analytical conditions.

Moreover, incorporation of somatic molecular testing integrated in pathology has a profound positive impact on attractiveness of pathology as a medical discipline in medical students who may decide to become pathologists. Furthermore, it has also important positive consequences in training and postgraduate education. The responsibility of incorporating molecular assays into routine pathology also requires deep understanding of the field by pathologists, not only for those directly involved in the workflow of the molecular tests.

An international strategy should be considered to give support to countries where molecular pathology is performed so far only in minimal extent, in order to face the scenario of increasing technological complexity and hardware requirements [25].

The issue of liquid biopsy (testing of circulating cell-free DNA released by the tumor—ctDNA) was not within the scope of this paper, which was exclusively focused on somatic molecular testing in tumor tissue. However, it is a very promising technique, regularly performed in many pathology departments across Europe. Although, at present, it is limited to a selected group of neoplasms such as lung cancer, its use will be probably extended also to other types of cancer in the near future.

## Technical issues

Technical issues are important to obtain correct results [26–30]. Pathology departments are responsible for providing the appropriate pre-analytical conditions which are crucial to get correct results. The control of pre-analytical factors is included also in the ISO15189 standard, which is to be followed to fulfill all conditions important for quality control of pathology departments and molecular laboratories. In Europe, the technical specifications for pre-analytics, issued by CEN (European Committee for Standardization) (https://www.cen.eu), should be applied. These are then translated into ISO international standards after 1 or 2 years. Involvement of pathologists in the entire process of molecular analysis ensures optimization of pre-analytical variables, such as cold ischemia time and optimal fixation.

Quality control is a very important issue. Pathology departments should demonstrate to be capable to perform somatic molecular testing in optimal conditions, at all steps of the technical process at the pre-analytical, analytical, and post-analytical levels. External quality control by accredited agencies is required.

Cold ischemia time is the period between tissue surgical removal and fixation. It is an important factor, since DNA, RNA, and proteins are altered during such period, with a clear impact on the results of testing using molecular techniques [30]. Tissue samples submitted to inappropriate fixation (too short, too long, inadequate concentration of fixative, etc.) may also yield incorrect results. Moreover, there are different protocols for paraffin-embedding as well as variations in this process that can affect the quality of the nucleic acids within the tissues.

As mentioned before, selection of the appropriate tissue block for molecular analysis is crucial in the light of tumor heterogeneity [31]. It is important to guarantee the optimal absolute number of neoplastic cells, as well as to quantify percentage of neoplastic cells in relation to non-neoplastic elements, such as inflammatory infiltrate, fibroblasts, and other cells of the tumor stroma. Presence of necrosis may contribute to difficulties to get correct results. Necrotic areas should be excluded from testing. If this is not possible, the amount of necrosis has to be quantified. Macro- or micro-dissection is frequently necessary to ensure the best quality of the tumor fragment for molecular analysis, and this can only be done by individuals with expertise in pathology. Occasionally, the initially selected tumor tissue fragment does not fulfill criteria for quality control, which leads to repetition of the procedure and selection of another tissue sample. Sometimes, the tissue does not fulfill quality requirements for NGS, and material may be submitted to analysis by an alternative single-gene approach. This workflow should follow standardized protocols to ensure appropriate turnaround times. Taking all these factors into account, a close integration of molecular testing into routine workflow of pathology departments is very important to guarantee the shortest turnaround time for interpretation of the results. This may have an important impact on initiation of appropriate treatment, namely, in patients with advanced disease.

## Conclusions

In this article, the importance of interpreting somatic molecular alterations of tumors in the light of morphology has been emphasized. A central role of pathology departments is necessary in the process of analysis and interpretation of somatic genomic alterations in cancer, not just as tissue providers for their interpretation outside this context. There are many reasons from the scientific, technical, and logistics points of view. The issue is important in terms of patient safety, since patients deserve clinically relevant interpretation of somatic alterations in cancer, being performed and interpreted under optimal conditions, quality control of the whole procedure, and optimal turnaround time.
